# Association between Personality Traits and Phubbing: The Co-Moderating Roles of Boredom and Loneliness

**DOI:** 10.3390/healthcare11060915

**Published:** 2023-03-22

**Authors:** Carla Abi Doumit, Diana Malaeb, Marwan Akel, Pascale Salameh, Sahar Obeid, Souheil Hallit

**Affiliations:** 1 School of Medicine and Medical Sciences, Holy Spirit University of Kaslik, Jounieh P.O. Box 446, Lebanon; 2 College of Pharmacy, Gulf Medical University, Ajman P.O. Box 4184, United Arab Emirates; 3 School of Pharmacy, Lebanese International University, Beirut 1083, Lebanon; 4 School of Medicine, Lebanese American University, Byblos 4504, Lebanon; 5 INSPECT-LB: Institut National de Santé Publique, Epidemiologie Clinique et Toxicologie-Liban, Beirut 1103, Lebanon; 6 Department of Primary Care and Population Health, University of Nicosia Medical School, Nicosia 2417, Cyprus; 7 Faculty of Pharmacy, Lebanese University, Hadat 1533, Lebanon; 8 School of Arts and Sciences, Social and Education Sciences Department, Lebanese American University, Jbeil 4504, Lebanon; 9 Applied Science Research Center, Applied Science Private University, Amman 11931, Jordan; 10 Research Department, Psychiatric Hospital of the Cross, Jal Eddib P.O. Box 60096, Lebanon

**Keywords:** phubbing, personality traits, boredom, loneliness, Lebanon

## Abstract

Phubbing is defined as the use of one’s phone while in a conversation, leading to a disconnection from interpersonal communications. This topic has become more interesting lately due to the increased use of smartphones worldwide and in Lebanon, where 86% of the population owns a smartphone. This study aims to assess the association between phubbing and personality traits with the moderating effects of loneliness and boredom proneness. This cross-sectional study was conducted between August–September 2020. 461 participants (18–29 years) filled out the survey. Higher open-mindedness was associated with less phubbing. Being married compared to single and higher open-mindedness were significantly associated with less phubbing. More boredom was significantly associated with more phubbing. The interaction extraversion by boredom was significantly associated with phubbing; this was significant at low levels of loneliness and boredom where extraversion was significantly associated with more phubbing. At moderate loneliness and high boredom and at high loneliness and boredom, extraversion was significantly associated with less phubbing. The interactions between boredom and loneliness and the other four personality traits did not show any significant association with phubbing. The current study highlights the importance of personality traits in influencing phubbing and might contribute to the preliminary assessment of phubbing. Healthcare professionals might be able to use the data of this study to implement appropriate smartphone use habits, as this would help patients improve their social relationships.

## 1. Introduction

Phubbing is defined as the use of one’s phone while in a conversation [1], leading to a disconnection from interpersonal communications. The term is a portmanteau of “phone” and “snubbing”. Phone snubbing is when one uses his/her phone during a social gathering rather than interacting and communicating with the people around. The “phubber” is the person who ignores and snubs others during a social gathering, while the “phubbee” is the person being ignored in such situations [2]. This topic has become of interest lately due to the increased use of smartphones worldwide and in Lebanon, where 86% of the population owns a smartphone [3]. Previous findings revealed that half of the interviewed participants were phubbed by their partners [4]. They also reported that this behavior led the “phubbee” to engage in the same act. As a result, emerging studies have been focusing on the factors that lead to phubbing and the influence phubbing has on our daily lives. For example, several studies showed that certain factors can result in increased phubbing behavior, such as fear of missing out (FoMO) [5], loneliness [6], boredom [2,6,7], internet and smartphone addiction, low self-control [8], and low self-esteem [9]. Moreover, phubbing behavior has been shown to disrupt interpersonal relationships as it decreases relationship satisfaction and communication quality [4,10,11]. 

Another factor that influences phubbing is problematic smartphone use (PMPU) [12]. PMPU is the inability to control mobile phone use and its online use. New research shows that this issue is correlated to negative emotionality, impulsivity, boredom proneness, dysregulated emotions, and sleep impairment [12]. Although the literature uses different terms for this phenomenon (mobile phone addiction, cellphone overuse, mobile phone dependence), it is described as a behavioral addiction based on symptoms of addictive behaviors. However, the term smartphone addiction cannot be used, as this type of addiction has not been included in the DSM-5. This differs from phubbing, as the latter is when one uses his/her phone while in a social gathering [12,13]. A positive relation between PMPU and phubbing has been shown by previous studies, where PMPU has been shown to increase phubbing [6,14].

Added to that, previous studies have shown relations between phubbing and personality traits [14,15,16]. Personality traits can be described using the five-factor model, consisting of five traits: openness to experience, conscientiousness, extraversion, agreeableness, and neuroticism/negative emotionality [17]. Openness is a personality dimension describing individual differences in appreciating new ideas, values, and behaviors. Conscientiousness is a trait that describes an individual who is punctual, alert, careful, and responsible. Extraversion reflects individuals that tend to be talkative, sociable, and friendly. Agreeableness is when someone is easygoing and agrees to what they are told without fighting for their opinion. Finally, neuroticism describes an individual who constantly feels negative emotions, sadness, and anxiety. It is important to know that an individual’s personality cannot be described by only one trait, but rather by a collection of traits [17]. One study showed a positive correlation between phubbing and high neuroticism, a negative correlation between phubbing and high openness to experience, and a lack of significant correlation with the other traits [14]. This is contradicted by a 2019 study that showed no correlation with openness and a positive correlation with conscientiousness [18]. The neutral correlation with agreeableness and extraversion, as well as the positive correlation with neuroticism were, however, consistent between the two studies [18].

Another factor correlated to phubbing is boredom proneness [2,6]. Boredom proneness, defined by Farmer and Sundberg in 1986 [19,20], is the tendency to experience boredom in several situations. It is defined by 3 terms: dissatisfaction, restlessness, and weariness [20]. Boredom proneness has been extensively studied throughout the recent years, with results showing its influence on our daily lives in a negative manner; boredom proneness is correlated to gambling behavior [21], anxiety and depression [20,22], increased technology use, decreased engagement in sports and hobbies, binge drinking behavior, increased risk of internet addiction [23], and phubbing behavior [2,6]. Studies have also shown its relation to poor interpersonal relationships, lower job satisfaction [19], and less overall happiness [6]. However, newer studies are starting to focus on the positive side of boredom proneness, as they suggest that boredom proneness keeps one moving forward and looking at newer goals [20]. It is a stimulant that motivates the individual to seek new experiences [20]. 

Similarly to boredom proneness, loneliness has had a positive correlation with phubbing [15]. Loneliness, as a concept and in its physiological form, can be defined as an unpleasant state an individual experiences due to a quantitative or qualitative deficit in his/her social connections [24]. Loneliness is separate from social isolation [25,26], which is an objective phenomenon in which an individual has no social connections. On the other hand, loneliness is subjective. One can be socially isolated yet not lonely [24,27]. Loneliness is divided into two parts: emotional loneliness and social loneliness. The former is the lack of a close relationship, while the latter is the absence of a larger social group such as friends and cousins [27]. Currently, statistics show that loneliness is on the rise. In fact, Cigna US loneliness index, done in 2020, revealed that 61% of adults experience loneliness, which is greater than in previous years [27]. Moreover, a survey revealed that people who constantly use social media experience more loneliness than the ones who do not [3]. Since loneliness is on the rise, it is interesting to correlate it to phubbing, which is also a rising phenomenon. While no previous studies have assessed the moderating effect of loneliness on the association between personality traits and phubbing behavior, several have shown a positive correlation between increased loneliness and phubbing behavior [14,15,16]. 

Phubbing behavior has been studied thoroughly recently; however, the literature still lacks evidence on many of its aspects. For instance, studies done on phubbing and personality traits showed no significant correlation between extraversion and phubbing [8,22]. However, extraversion is a known correlate of problematic phone use [28], which, in turn, is associated with phubbing [29]. The literature also lacks a significant correlation between agreeableness and phubbing. As mentioned before, being agreeable means being friendly and easy-going, yet it is still unknown whether agreeable individuals are prone to phubbing behavior or not [2]. Other than that, it has been demonstrated that phubbing behavior affects our daily lives by negatively influencing social relationships [29]. Therefore, knowing the predictors of this behavior will help psychologists, social workers, and physicians figure out ways to treat this emerging issue. Moreover, the literature shows evidence of an association between phubbing and boredom proneness [6,7] and phubbing with loneliness [16]. However, to our knowledge, no literature has been found to associate these variables as moderators between phubbing and personality traits. The reason for using loneliness and boredom proneness as moderators, not mediators, is to conclude if the stated variables affect the direction and strength of the relation between phubbing and personality traits. In other words, we are interested in knowing if loneliness and boredom proneness modify the relationship between personality traits and phubbing. Therefore, a mediating effect between the variables would not be beneficial, as it would establish an indirect effect between them, not an interaction. Added to that, while rich literature exists over phubbing, none of the research was applied on the Lebanese population. The data could be valuable for ethnic and cultural comparison. 

This study aims to assess the association between phubbing behavior and personality traits with the moderating effects of loneliness and boredom proneness. In this study, we hypothesize that higher extraversion would be significantly associated with less phubbing. We also hypothesize that high levels of boredom proneness and loneliness in an individual would significantly correlate to more phubbing. 

## 2. Materials and Methods

The Psychiatric Hospital of the Cross Ethics and Research Committee approved the study protocol (HPC-033-2020). The purpose and requirements of the study were explained to each participant, and filling out the form and submitting it online consented that the person approved to participate in the study and. This was considered equivalent to obtaining a written informed consent. 

This is a cross-sectional study done by using a sample of members of the community aged 18 to 29 years old during August and September 2020. This age range was chosen because this category tends to use mobile phones more than others [3]. We employed an anonymous, self-administered questionnaire produced using Google Forms due to the COVID-19 pandemic making in-person questionnaires unfeasible. Using the snowball technique, the link was disseminated among the participants and delivered to all districts/governorates in Lebanon via students and social networks. Participants with a mobile phone between the ages of 18 and 29 years were invited to join and requested to send the link. Those that declined to fill out the questionnaire were not included [30]. 

According to the G-power software and based on an effect size f^2^ = 2%, an alpha error of 5%, a power of 80%, and a total of 12 factors entered in the multivariable analysis model, a minimum of 395 participants was deemed to be necessary. At the end of the data collection, 561 individuals responded to the questionnaire.

The mean age of the participants was 22.25 ± 2.87 years, with 70.9% being females. The vast majority of participants (94.4%) had a university-level education. The participants’ household crowding index had a mean of 1.08 ± 0.61, whereas the phubbing score was 41.36 ± 16.94. Other characteristics of the participants are displayed in Table 1.

Participants were given a self-administered, anonymous questionnaire with closed-ended questions. The questionnaire was available in English and Arabic, and it took about 25–30 min to complete. The questionnaire underwent a forward and back translation process by a medical student and mental health professional and consisted of different sections. The first part identified the participant’s socio-demographic characteristics: age, gender, educational level, and household crowding index (quotient of household residents over the number of rooms excluding the kitchen and bathroom [31]). The following scales were used for the second part of the questionnaire: 

Phubbing has been assessed using the Generic Scale of Phubbing (GSP) [8], which is a 15-item scale divided into 4 parts. Validated in Lebanon [32], it includes 4 items about detachment from one’s phone, 4 items about interpersonal conflicts, 4 items about self-isolation, and 3 items about using one’s phone to escape social situations. An example of a sample item would be: “I feel anxious if my phone is not nearby”. Responses were measured on a seven-point scale from 1 to 7, where 1 signifies that the participant never does phubbing and 7 indicates very regular phubbing. The Cronbach’s alpha for this study’s phubbing scale was 0.929.

To examine the big five personality domains and its facets, the Big Five Inventory (BFI-2) [33] has been used. This questionnaire, validated in Arabic [34], consists of 60 items relating to different personality constructs and domains. Assertiveness and activity are handled in the extraversion domain, in which it is asked if one is “outgoing and sociable”. Altruism and compliance are tested for the agreeableness domain, in which it is asked if one “treats others with respect”. Moreover, order and self-discipline are tackled in the conscientiousness domain, in which it is asked if “one likes to keep things in order”. In the negative emotionality domain, anxiety and depression are tackled by asking if one “can be tense”, and “is emotionally stable, not easily upset”. Finally, aesthetics and ideas are tackled in the openness domain by asking questions about creativity: “has difficulty imagining things/has little creativity”. Results have been measured using a 5-point Likert scale ranging from strongly disagree at 1 point to strongly agree at 5 points, where higher scores indicate higher defectiveness in that specific domain. The Cronbach’s alpha values for each personality trait are as follows: extraversion (0.649), agreeableness (0.621), conscientiousness (0.698), negative emotionality (0.691), and open mindedness (0.625).

Boredom has been assessed using the short form [35] of the Boredom Proneness Scale (BPS) [19]. The scale is an 8-item scale assessing the tendency to experience boredom in different situations. The answers were measured on a 7-point Likert scale ranging from 1 (strongly disagree) to 7 (strongly agree). Higher scores indicate higher boredom proneness. In this study, the Cronbach’s alpha is 0.811.

Validated in Lebanon [36], the five-item modified version of the Jong-Gierveld Loneliness Scale has been used to assess the subjective loneliness of participants [36]. One of the items on the scale is: “I often feel rejected”. A binary system was implemented and a “yes” equals one and a “no” equals a zero, so higher scores would indicate higher loneliness. This study generates a Cronbach’s alpha value α = 0.815.

The 23rd version of the SPSS software was used to perform some parts of the data analysis. Cronbach’s alpha values were recorded for reliability analysis of all scales and subscales. Calculations of skewness and kurtosis were used to validate the phubbing score, with values for these between −2 and +2 being considered adequate in order to prove the normal univariate distribution [37]. These requirements strengthen the assumption of normality in a sample of this size (>300) [38]. For bivariate analysis, students and the Pearson test were used to compare 2 means and correlate 2 continuous variables respectively. This was followed by the use of the ENTER method to conduct linear regression, with the phubbing score being the dependent variable and independent variables with a *p* < 0.2 in the bivariate analysis being included [37]. Assumptions of multicollinearity were assessed via the calculation of the Variance Inflation Factor (VIF), which should be below 4 and tolerance values above 0.2 [38]. After that, interactions between personality traits and boredom/loneliness were processed using PROCESS SPSS Macro version 3.4 model 1 [39]. A value *p* < 0.05 was considered significant. 

## 3. Results

### 3.1. Bivariate Analysis

The results of the bivariate analysis of factors associated with phubbing are summarized in Table 2 and Table 3. A higher mean generic scale of phubbing score was significantly found more in single participants as compared to married ones. In addition, more boredom, more loneliness, and higher negative emotionality were significantly associated with more phubbing. Higher extroversion, higher agreeableness, higher conscientiousness, and higher open-mindedness were significantly associated with less phubbing.

### 3.2. Multivariate Analysis

The normal P-P plot of regression standardized was residual, and the scatterplot, as well as other statistical assumptions (outliers, normality, linearity, and homoscedasticity) were verified. The results of the linear regression, using the ENTER model and taking the phubbing scale as the dependent variable, showed that being married compared to single (Beta = −5.01) and higher open mindedness (Beta = −0.42) were significantly associated with less phubbing. More boredom (Beta = 0.21) was significantly associated with more phubbing, while negative emotionality showed a positive borderline result (B = 0.23), as well as loneliness (B = 0.31) (Table 4).

### 3.3. Interaction Analyses

The interaction extraversion by boredom was significantly associated with phubbing (Table 5); this was significant at low levels of loneliness and boredom, where extraversion was significantly associated with more phubbing (Beta = 0.50). At moderate loneliness and high boredom (Beta = −0.41) and at high loneliness and boredom (Beta = −0.54), extraversion was significantly associated with less phubbing (Table 6). The interactions between boredom and loneliness and the other four personality traits did not show any significant association with phubbing (Table 5).

## 4. Discussion

This study aimed to establish if there is a relationship between personality traits and phubbing, and if loneliness and boredom proneness have moderating effects. In this study, we were able to reveal a significant association between marital status, boredom proneness, and open-mindedness with phubbing behavior. Moreover, the interaction between extraversion and boredom was significantly associated with phubbing; in people with moderate to high boredom proneness levels, high extraversion was significantly associated with less phubbing behavior.

Our first hypothesis was that higher extraversion is significantly associated with less phubbing. Our hypothesis is based on the fact that highly extroverted individuals have rich interpersonal relationships and enjoy being around people [40]. In this study, similarly to previous ones [40,41], no significant association has been established between high extraversion and phubbing. However, our results show that boredom proneness is a moderator to extraversion and phubbing. To elaborate, in people with moderate to high levels of boredom proneness, higher extraversion leads to less phubbing. This can be explained by the fact that highly extroverted individuals are sociable beings who are more satisfied with their social relationships [40]. This indicates that they might fill their boredom with socializing instead of using their phones. 

We also believe that high levels of boredom proneness and high levels of loneliness are both positively correlated to more phubbing. In our study, a significant association has been established only between boredom proneness and phubbing. Our results show that both variables are positively correlated. This is in line with previous research since the same association has been established [6,7]. Other studies reveal that boredom is associated with problematic smartphone use [41,42,43], which is an indicator of phubbing [18]. This is explained by the fact that smartphone use and phubbing lead to relaxation, entertainment, sociability, and interpersonal connectivity [44], which could be a coping mechanism for boredom. However, the association between loneliness and phubbing is still controversial. While our results show no significant association between the two variables in the multivariable model and are in line with a previous study [6], different studies show a positive association between both variables [14,18]. This association can be explained by the fact that lonely individuals tend to mask their loneliness and embarrassment by using their phones [45]. They can also feel more connected, which would alleviate their loneliness [46].

Finally, high openness is defined by being open to new experiences, imaginative, curious, creative, free, and highly intellectual [17]. In our study, higher openness is correlated to less phubbing behavior. This is consistent with previous results that have shown how openness negatively predicts phubbing behavior [47]. Another study shows no association between phubbing and openness [18]. Our results are explained by the fact that open-minded individuals have the curiosity to explore their current environment and seem more interested in face-to-face interactions to fulfill their needs. 

Another finding is that being married is associated with less phubbing compared to being single. While no previous literature has discussed this issue, it has been shown that married couples experience less loneliness and less boredom, as they are distracted by the responsibilities of being married and have probably filled the gap of loneliness by marriage [44]. 

### Limitations

Data was collected through a convenient sampling, leading to a sample composed predominantly of female, single, highly educated participants; thus, results might not be generalizable to the whole population. The refusal rate is unknown, which precludes concluding about the prevalence of different concepts due to the selection bias. Questions might have been over or underestimated by responders, leading to a possible information bias. A residual confounding bias is possible since not all factors associated with phubbing were included in the questionnaire; the determination coefficient is low, indicating that the variables entered in the model explain 25% of the dependent variable only. Furthermore, the boredom scale has not been validated in Lebanon yet. Finally, we have done regression modeling using a convenience sample; therefore, we are implicitly assuming the probability of a person being included in the sample depends only on the variables included in our regression model, which might not be true. We are also assuming equal-probability sampling within the post-stratification cells determined by the predictors in our regression. Moreover, the cross-sectional study design does not allow causality-related conclusions. For these reasons, our results might lack robustness and should be interpreted with caution. Future studies taking into account those limitations should be conducted. Another suggestion could be to directly interview children about their relationships with parents, as well as exploiting parents’ point of view.

## 5. Conclusions

In this study, we have been able to show a significant association between marital status, boredom proneness, and open-mindedness with phubbing behavior. Moreover, in people with moderate to high boredom proneness levels, high extraversion is significantly associated with less phubbing behavior. Based on the results of this study, and as smartphone use and phubbing behavior continue to rise, clinical psychologists can address this issue while focusing on the moderating effects of boredom and loneliness. Moreover, while dealing with psychiatric patients, healthcare professionals might be able to implement appropriate smartphone use habits, as this would help patients improve their social relationships. 

## Figures and Tables

**Table 1:** Sociodemographic characteristics of the study sample (N = 461).

	Frequency (%)
**Gender**	data
Male	134 (29.1%)
Female	327 (70.9%)
**Marital status**	
Single	421 (91.3%)
Married	40 (8.7%)
**Education level**	
School education	26 (5.6%)
University education	435 (94.4%)
	**Mean ± SD**
**Age (in years)**	22.25 ± 2.87
**Household crowding index**	1.08 ± 0.61

**Table 2:** Bivariate analysis of categorical variables associated with the phubbing score.

	Generic Scale of Phubbing	*p*-Value
	Mean ± SD	
Gender		
Male	40.13 ± 16.36	0.382
Female	41.86 ± 17.16	
Marital status		
Single	41.85 ± 17.03	0.032
Married	36.15 ± 15.14	
Education level		
School education	41.38 ± 16.38	0.853
University education	41.36 ± 16.98	

**Table 3:** Correlation matrix between the variables.

	P	B	L	E	A	C	NE	OM	HCI	Age
Phubbing (P)	1									
Boredom Proneness (B)	0.410 ^c^	1								
Loneliness (L)	0.281 ^c^	0.369 ^c^	1							
Extraversion (E)	−0.233 ^c^	−0.060	−0.182 ^c^	1						
Agreeableness (A)	−0.175 ^c^	0.071	−0.045	0.363 ^c^	1					
Conscientiousness (C)	−0.216 ^c^	−0.052	−0.052	0.437 ^c^	0.512 ^c^	1				
Negative emotionality (NE)	0.247 ^a^	0.223 ^c^	0.349 ^c^	−0.372 ^c^	−0.274 ^c^	−0.298 ^c^	1			
Open-mindedness (OM)	−0.189 ^a^	0.079	−0.016	0.547 ^c^	0.511 ^c^	0.532 ^c^	−0.096 ^a^	1		
Household crowding index (HCI)	−0.055	−0.046	−0.064	−0.046	−0.014	−0.031	−0.041	−0.097 ^a^	1	
Age	−0.013	0.101 ^a^	−0.093 ^a^	0.203 ^c^	0.184 ^c^	0.147 ^b^	−0.182 ^c^	0.103 ^a^	−0.058	1

^a^*p* < 0.05; ^b^ *p* < 0.01; ^c^ *p* < 0.001.

**Table 4:** Linear regression of factors associated with the generic scale of phubbing score taken as the dependent variable.

Variable	Unstandardized Beta	Standardized Beta	*p*	95% CI		Tolerance	VIF
Marital status (married vs. single *)	−5.01	−0.08	**0.043**	−9.86	−0.17	0.965	1.036
Boredom Proneness	0.21	0.38	**<0.001**	0.16	0.26	0.815	1.227
Loneliness	0.31	0.08	0.075	−0.03	0.65	0.755	1.324
Extraversion	−0.11	−0.04	0.445	−0.39	0.17	0.572	1.748
Agreeableness	−0.19	−0.07	0.174	−0.45	0.08	0.628	1.592
Conscientiousness	−0.16	−0.06	0.221	−0.41	0.09	0.595	1.680
Negative emotionality	0.23	0.09	0.058	−0.01	0.46	0.697	1.435
Open-mindedness	−0.42	−0.16	**0.007**	−0.72	−0.12	0.491	2.035

* Reference group; numbers in bold indicate significant *p* values; Determination coefficient = 0.266.

**Table 5:** Interaction analyses.

Model 1: Interaction Personality Traits by Loneliness and Bordom on Phubbing.				
Personality Trait	Beta	t	*p*	95% CI
Extraversion				
Interaction 1 by loneliness	−0.03	−1.33	0.185	−0.07; 0.01
Interaction 2 by boredom	−0.01	−2.63	**0.009**	−0.02; −0.003
Agreeableness				
Interaction 1 by loneliness	−0.04	−1.64	0.101	−0.09; 0.01
Interaction 2 by boredom	0.01	1.53	0.126	−0.002; 0.02
Conscientiousness				
Interaction 1 by loneliness	−0.02	−0.75	0.451	−0.06; 0.03
Interaction 2 by boredom	−0.01	−1.81	0.072	−0.02; 0.001
Negative emotionality				
Interaction 1 by loneliness	0.04	1.90	0.058	−0.001; 0.08
Interaction 2 by boredom	−0.01	−1.07	0.285	−0.02; 0.01
Open-mindedness				
Interaction 1 by loneliness	−0.03	−1.28	0.201	−0.08; 0.02
Interaction 2 by boredom	0.001	−0.08	0.937	−0.01; 0.01

Numbers in bold indicate significant *p* values.

**Table 6:** Conditional Effect of Focal Predictor (Extraversion) at Values of the Moderator Variables (Loneliness and Boredom).

Loneliness	Boredom	Coefficient	SE	*t*	*p*	95% CI
Low (=9.17)	Low (=77.28)	0.50	0.23	2.16	**0.032**	0.04; 0.95
Low (=9.17)	Moderate (=107.48)	0.11	0.17	0.65	0.516	−0.23; 0.45
Low (=9.17)	High (=137.68)	−0.27	0.22	−1.24	0.214	−0.71; 0.16
Moderate (=13.67)	Low (=77.28)	0.36	0.23	1.60	0.11	−0.08; 0.81
Moderate (=13.67)	Moderate (=107.48)	−0.02	0.14	−0.15	0.883	−0.31; 0.26
Moderate (=13.67)	High (=137.68)	−0.41	0.18	−2.25	**0.025**	−0.76; −0.05
High (=18.16)	Low (=77.28)	0.23	0.27	0.87	0.385	−0.29; 0.75
High (=18.16)	Moderate (=107.48)	−0.15	0.18	−0.86	0.393	−0.51; 0.20
High (=18.16)	High (=137.68)	−0.54	0.19	−2.81	**0.005**	−0.92; −0.16

Numbers in bold indicate significant *p* values.

## Data Availability

The authors do not have the right to share any data information as per the ethics committee rules and regulations.
